# SOCS Proteins in Macrophage Polarization and Function

**DOI:** 10.3389/fimmu.2014.00357

**Published:** 2014-07-28

**Authors:** Heather M. Wilson

**Affiliations:** ^1^Division of Applied Medicine, Institute of Medical Sciences, University of Aberdeen, Aberdeen, UK

**Keywords:** suppressor of cytokine signaling proteins, macrophage, M1, M2, inflammation

## Introduction

Macrophages were initially described as “big eaters” due to their phagocytic nature. It is now clear that macrophages have many diverse functions not only in innate immunity and tissue homeostasis but also in metabolism, development, and regeneration. Macrophage functions are driven largely by tissue-derived and pathogenic microenvironmental stimuli that help them adapt to changing conditions within tissues and tailor an appropriate response. The heterogeneity of macrophages has resulted in their classification into subtypes based on their phenotype and function (1). One major classification, based on function, is M1 and M2 macrophages, with destructive and healing properties, respectively (2, 3). As imbalances between M1 and M2 states have been observed in a number of diseases, an understanding of the molecular mechanisms, signaling pathways, and transcription factors controlling their polarization has obvious therapeutic implications. Recent studies have established strong potential for suppressor of cytokine signaling (SOCS) proteins to regulate M1 and M2 macrophage polarization (4–7). Here, the focus will be on the evidence for this, and the consequences of altered SOCS expressions on macrophage function in health and disease. Overall it is proposed that a high SOCS1 to SOCS3 ratio could be a potential marker for M2 macrophages while high SOCS3 expression is associated with M1 cells.

## SOCS Proteins

Suppressor of cytokine signaling proteins are a family of intracellular cytokine-inducible proteins, consisting of eight members (CIS and SOCS1–SOCS7) (8, 9). SOCS1 and SOCS3 are most widely characterized regarding their roles in shaping M1 and M2 macrophage polarization (4–6). They show low expression in resting macrophages, but are rapidly induced on activation. All SOCS family proteins contain an Src homology 2 (SH2) domain, a variable length amino-terminal domain and a conserved carboxy-terminal SOCS box motif that interacts with ubiquitin–ligase machinery (8, 9). SOCS are induced by a variety of stimuli that cause M1 and M2 activation, including cytokines, toll-like receptor (TLR) ligands, angiotensin II, immune complexes, and high glucose (9). The most studied signaling pathway regulated by SOCS is JAK/STAT activation. SOCS negatively regulate JAK/STAT signaling through association with key phosphorylated tyrosine residues on JAK proteins and/or cytokine receptors, and by degradation of signaling molecules mediated via the ubiquitin–proteasome pathway (8, 9). SOCS1 and SOCS3 contain a kinase inhibitory region (KIR) that directly suppresses JAK tyrosine kinase activity. SOCS proteins also influence ERK (10), PI3K (11), Notch (12), MAPK (13), and NF-κB (14) signaling cascades that directs M1 and M2 functions.

### SOCS1

**Figure 1:**
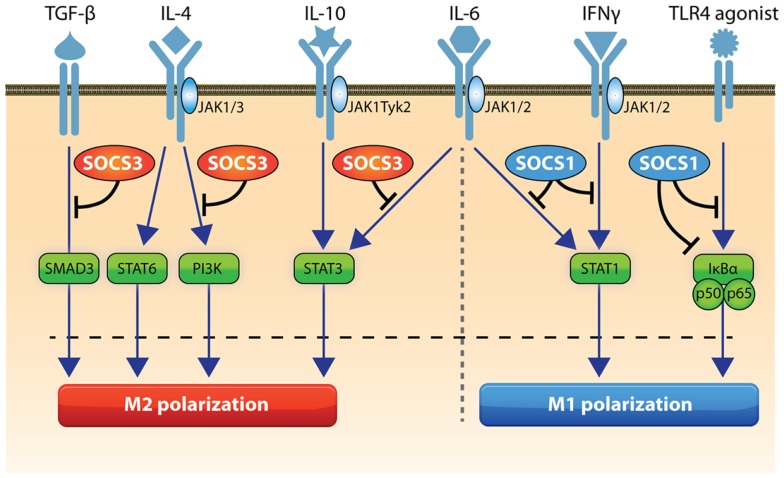
**Role of SOCS1 and SOCS3 in macrophage activation**. STAT1 and NFκB drive M1 polarization and SOCS1 can inhibit these pathways. SOCS3 can regulate TLR signaling and inhibits IL-6-induced STAT3 activation and SMAD3 and PI3K activity to action an appropriate destructive effect. STAT3, STAT6, and PI3K can drive M2 activation and SOCS3 inhibits STAT3 and PI3K. Pathways that trigger SOCS1 in macrophages include STAT1 and NFκB, while SOCS3 expression can be induced by STAT3, NFκB, NOTCH1, PI3K, and MAP kinase activation.

SOCS1 regulates M1-macrophage activation by inhibiting the interferon gamma-induced JAK2/STAT1 pathway and TLR/NF-κB signaling (9, 15) (Figure 1). To suppress the latter pathway, SOCS1 binds to the p65 subunit of NF-κB and the TLR adaptor molecule Mal/TIRAP as well as IRAK, facilitating its ubiquitin-mediated proteolysis via ubiquitin ligases recruited by the SOCS box (8, 14–17). SOCS1 indirectly inhibits TLR4 signaling through secondary mechanisms targeting IRF3 and IFN-β induced JAK/STAT pathways (18, 19). Thus, SOCS1 mediates a negative feedback mechanism during TLR4 signaling, via control of both MyD88-dependent and MyD88-independent signaling. SOCS1-deficient mice succumb to severe systemic autoimmune and inflammatory disease (14, 16) and their M1-macrophages display an increased capacity to kill intracellular bacterial pathogens, presumably due to unrestrained IFN-γ/STAT1 and p65 signaling. In line with this, SOCS1 knockout or knockdown M1-activated macrophages show enhanced levels of IL-6, IL-12, MHC class II, and nitric oxide suggesting SOCS1 sustains the properties of M1 macrophages at a less destructive level to prevent overshooting inflammatory responses (4, 18). This explains why SOCS1 promoter hypermethylation, which results in loss of SOCS1 expression leads to enhanced secretion of lipopolysaccharide (LPS)-induced pro-inflammatory cytokines (20). Micro RNA-155 (miR-155) is a critical regulator of innate immunity and TLR signaling (21–23); miR-155 targets and degrades SOCS1 in M1-activated macrophages (21), thus miR-155 induction during activation serves to maximize and extend the inflammatory process.

SOCS1 also regulates M2 macrophage polarization. Expression of macrophage SOCS1, but not SOCS3, is strongly upregulated in an M2 polarizing environment *in vitro* and *in vivo*, where it has an important role in acquisition of M2 functional characteristics, such as a high arginase I/low inducible nitric oxide synthase (iNOS) expression ratio (4). Strikingly, this contrasts with macrophages infiltrating an *in vivo* inflamed M1-activating environment, where macrophages with enhanced SOCS3 but not SOCS1 expression are prominent (5). This suggests that exclusive upregulation of SOCS1, or indeed, a high SOCS1/SOCS3 expression ratio, has potential as a useful and additional *in vivo* biomarker for M2 (see later). Arginase I expression, as an M2 macrophage marker, can be mediated via activation of either STAT6 (24) or PI3K (25). SOCS1 is important in controlling PI3K activity, supporting a mechanism for regulating arginase I expression in M2 cells; SOCS1 also regulates STAT6 phosphorylation (26). Following activation, SOCS1 knockdown or SOCS1-deficient macrophages show a reciprocal upregulation of SOCS3 expression. SOCS3 inhibits PI3K activation (27), and so the expression of high SOCS1 and low SOCS3 in M2 macrophages could result in greater PI3K activity and more arginase I induction in these cells. An elevated expression of SOCS1 is important for the arginase I-induced suppressive nature of M2 macrophages that attenuate lymphocyte proliferation (28). Moreover, siRNA-mediated knockdown of SOCS1 results in the induction of iNOS in IL-4-pretreated cells stimulated with IFN/LPS (4). Thus, SOCS1 regulates the iNOS/arginase I expression ratio in both M1 and M2 macrophages and helps fine-tune key signaling pathways to mount an appropriate response to changes within the microenvironment.

### SOCS2

An important role for SOCS2 in driving M2 polarization and limiting M1 polarization has been shown, with IL-4 activation of macrophages, resulting in enhanced SOCS2 expression (27). Macrophages from SOCS2^-/-^ mice display increased secretion of IFN-γ, IL-1β, and TNF-α in response to LPS in parallel to an increased pro-inflammatory cytokine mRNA expression (29). These BMDMs have higher basal levels of p65–NF-κB compared with macrophages from wild-type mice (29). In another study, SOCS2-deficient macrophages were hyper responsive to IFN-γ, produced more NO and dealt with infection more efficiently (30). SOCS2 has also been described as a feedback inhibitor of TLR-induced activation in dendritic cells (31).

### SOCS3

In contrast with SOCS2, a key role for SOCS3 in M1 polarization is proposed (Figure 1). The majority of macrophages activated within an *in vivo* pro-inflammatory conditioning environment show strong upregulation of SOCS3 expression and this cell population co-express the M1 marker, iNOS (5, 6). Without SOCS3, both human and rodent macrophages have a reduced ability to develop pro-inflammatory features but instead display immunoregulatory characteristics (5, 6). Notably, mice with a targeted deletion of SOCS3 in macrophages and neutrophils demonstrate a reduced IL-12 response and succumb to toxoplasmosis (32). SOCS3 binds to and inhibits gp130-related cytokine receptors and consequently this abrogates IL-6-induced STAT1 gene expression and IL-6-induced STAT3 anti-inflammatory effects (33–35). Therefore, in SOCS3-deficient macrophages, IL-6 signals in a similar manner to the immunosuppressive cytokine IL-10, through prolonged STAT3 activation and dampening of LPS signaling (33). As a result, mice deficient in SOCS3 in myeloid cells are resistant to endotoxic shock (35) with reduced production of pro-inflammatory cytokines. However, one report in the same mice suggests SOCS3 deficiency promotes M1 macrophage activation in spite of enhanced STAT3 activation (7). The reasons for this discrepancy in findings are unclear but could relate to differences in dose and purity of the LPS used in the different studies, as well as and the genes and time-points analyzed after macrophage activation (7, 35). Moreover, the conflicting results for the role of SOCS3 in M1 polarization in isolated macrophages *in vitro* (5–7) could result from the different technologies and species used (siRNA-mediated knockdown in rat and human macrophages, which avoids the risk of compensatory effects of other SOCS genes (5, 6) versus cells from macrophage-specific SOCS3 knockout mice) (7). Resolving these issues should establish the importance of SOCS3 in modulating macrophage function *in vivo*.

Studies of SOCS3-deficient macrophages confirm that SOCS3 positively regulates TLR4 signaling and M1 activation by inhibition of IL-6R-mediated STAT3 activation, as well as TGF-β-mediated SMAD3 activation, which is critical for the negative regulation of TLR-induced TNF-α and IL-6 production (5, 6, 33, 36). Since SOCS3 blocks PI3K that feeds and inhibits TLR responses, this could be an alternative mechanism by which SOCS3 augments TLR signaling in M1 macrophages (6). Forced activation of Notch signaling enhances both M1 polarization and anti-tumor activity via SOCS3 induction (12). In line with this, macrophage-specific SOCS3 knockout animals are resistant to tumor transplantation due to reduced secretion of tumor-promoting TNF-α and IL-6, together with elevated MCP2/CCL8 that is anti-tumorigenic (37).

Regulation of SOCS3 in innate cells influences downstream T cell fates. The presence of SOCS3 in macrophages is important in fine-tuning downstream T effector cell priming due to both influences in expression of presenting molecules and altered secretion of T cell polarizing cytokines (6, 7). Mouse SOCS3-deficient dendritic cells display an analogous reduced potential to drive T effector cell responses and a tolerogenic phenotype as a result of enhanced TGFβ production and expansion of Foxp3-positive regulatory T cells (38). These dendritic cells reduce the severity of experimental autoimmune disease. Therefore, regulation of intracellular signaling pathways by SOCS3 in innate cells is critical for the decision of adaptive responses such as T cell fates. The depletion of macrophage SOCS3 in a clinical situation would thus be predicted to dampen both pro-inflammatory innate and adaptive immune responses.

The above studies suggest that macrophage SOCS3 is associated with M1 macrophages and pro-inflammatory responses and is a potential therapeutic target in inflammatory diseases. However, a word of caution should be introduced as this may not be the case in all inflammatory conditions. In diseases, where STAT3 activation exerts a profound inflammatory and pathogenic response (39, 40) then the effects of SOCS3 targeting may not be beneficial. For example, in an IL-1/STAT3 model of chronic arthritis where SOCS3 was deleted in hematopoietic and endothelial cells, animals exhibited more severe disease. Thus, the pathology needs first to be assessed before SOCS3 manipulation as a therapy is considered (37).

## Macrophage SOCS Expression and Pathology

The heightened expression of macrophage SOCS1 and SOCS3 proteins have been demonstrated in many pathologies *in vivo* where this has been proposed, through the molecular mechanisms described above, to enhance or inhibit pathogenesis.

### SOCS and glomerulonephritis

Macrophages are an important feature in glomerulonephritis pathology. Macrophages infiltrating inflamed glomeruli in experimental models are rapidly polarized to express either SOCS1 or SOCS3, but rarely both, with most exclusively expressing SOCS3 (5, 6). The proportion of these SOCS3-expressing macrophages correlates strongly with the severity of immune-mediated injury. Local delivery of IL-4 to inflamed glomeruli has a major effect on reducing the number of SOCS3-expressing glomerular macrophages, and this is reflected by a decrease in the severity of nephritis, supporting a role for SOCS3 in driving M1-mediated injury (5).

### SOCS and atherosclerosis

Human atherosclerotic plaques exhibit a high expression of macrophage SOCS1 and SOCS3 in unstable inflammatory shoulder regions as compared to stable fibrous area (41). SOCS1 and SOCS3 expression is increased in aortic lesion macrophages from apoE(−/−) mice (42). In human tissue, the percentages of SOCS1-positive, M2 macrophages are decreased in morphologically stable atherosclerotic plaques, whereas percentages of SOCS3-positive, iNOS positive, macrophages are increased in unstable, rupture-prone plaques, suggesting targeting macrophage SOCS3 would be beneficial to dampen inflammation and plaque vulnerability (43). The differing expression ratio of SOCS1:SOCS3 in atherosclerotic plaques again suggests that the ratio could be an indicator of the inflammatory status of human macrophages *in vivo*. SOCS1 was atheroprotective in mouse models (44) while the absence of macrophage SOCS3 of apoE(−/−) mice attenuates disease, confirming a causal link between macrophage SOCS3 and atherosclerosis (45).

### SOCS and inflammatory bowel disease

Beneath the gut epithelia, lamina propria macrophages phagocytose bacteria and maintain an M2 phenotype in the steady state. Approximately 10% of these macrophages express SOCS3 in healthy individuals, whereas in inflammatory bowel disease (IBD) patients this increases to 40%, again suggesting SOCS3 expression relates to M1-activated macrophages (46). Peroxisome proliferator-activated receptor-γ (PPARγ) agonists demonstrate efficacy in ameliorating intestinal inflammation associated with IBD. PPARγ expression is upregulated in M2 but not M1 macrophages. In macrophages lacking PPARγ, a significant upregulation of SOCS3 was noted and this could be important if treating IBD with PPARγ agonists (47).

### SOCS and tumors

In human tumors, SOCS3 expression identifies macrophages with enhanced tumor killing, whereas SOCS1 expressing macrophages (M2) favor tumor survival (48). Macrophage-specific deletion of SOCS1 leads to reduced susceptibility to melanoma growth and colon carcinogenesis through increased anti-tumor responses (49) and a switch to M1 polarization of tumor-associated macrophage. In contrast, mice with a macrophage-specific deletion of SOCS3, subcutaneously implanted with melanoma cells, did not show a difference in tumor size, although the number of metastasis increased in these mice (37). These SOCS3-deficient macrophages produce less IL-6 and TNF-α upon stimulation with tumor lysates due to aberrant STAT3 activity, again showing a positive link of SOCS and macrophage polarization (37).

### SOCS and obesity

SOCS3 restrains macrophage responses to IL-6 and leptin that are systemically upregulated in obesity (50). SOCS1 inhibits insulin signaling and macrophage cytokine secretion, resulting in insulin sensitivity in spite of an obese state (17). Moreover, an increase in SOCS1 expression in mouse macrophages inhibits LPS- and palmitate-induced TLR4 signaling and in so doing prevents systemic inflammation and hepatic insulin resistance (17).

## Conclusion and Perspectives

Given the broad role of SOCS in regulating macrophage functions in health and disease, the modulation of macrophage-specific SOCS1 and SOCS3 expression provides new opportunities for therapeutic manipulation of immune and inflammatory responses. However, it is not only macrophages that are affected by SOCS proteins. Other cell types upregulate and react to SOCS proteins to shape cellular functions. Targeting SOCS specifically in macrophages is therefore important as an efficient means of changing the inflammatory response.

## Conflict of Interest Statement

The author declares that the research was conducted in the absence of any commercial or financial relationships that could be construed as a potential conflict of interest.
